# Supplementary material for: Comparison of human population receptive field estimates between scanners and the effect of temporal filtering
Source: F1000Res. 2020 Feb 26;8:1681. Originally published 2019 Sep 24. [Version 2] doi: 10.12688/f1000research.20496.2 (PMC6913234; doi:10.12688/f1000research.20496.2)
Supplement: Peer review history

# Peer review: Reviewer response for version 1

**DOI:** 10.5256/f1000research.22528.r56563

**Published:** 2019-12-12

**Authors:** Paola Binda

**Affiliations:** 1 Faculty of Medicine, University of Pisa, Pisa, Italy

pRF mapping provides a quantitative description of cortical representations, including the spatial representation in visual cortical areas. While pRF parameters are extensively used to quantitatively compare across subjects and conditions, we are well aware that such parameters depend on both neural factors and on nuisance variables that are related to the dynamics of BOLD. However, we lack a precise understanding of how such nuisance variables might affect each of the pRF parameters. Morgan & Schwarzkopf make an important effort in this direction and test the impact of static SNR (affected by MR field strength) and temporal SNR (affected by temporal filtering) on spatial pRF parameters in visual cortical areas.

 I have no major concerns with the study and only offer few suggestions that hopefully will increase the usability of the reported results:

1. It would be useful to explicitly describe one (or more) models that predict changes of pRF parameters with SNR.
  I feel one intuitive (and probably wrong) model invokes a non-linear thresholding effect. The logic is, approximately, as follows: when BOLD responses are stronger, each voxel responds to a larger range of stimulus position and the pRF size is consequently increased. Is this one of the hypotheses that the study intends to test (and falsify)?
  This particular model would predict that beta values (indexing BOLD response amplitude) and pRF size estimates correlate positively. Since both indices were collected here, could the authors measure this (lack of) correlation?
  In addition, this model would incorrectly predict larger pRF size estimates at higher MR fields, under the assumption that response amplitude is positively related to SNR. Could the authors report whether this assumption (larger beta-values in the higher SNR, i.e. 3T, dataset) is correct for their specific dataset? 
  Also, I wonder whether one could also expect variations of the other pRF parameter (pRF position), e.g. in the form of an attraction towards the fovea / screen borders / horizontal and vertical meridians. The text reports relatively high correlations between pRF center parameters at 1.5 and 3T, but this does not exclude systematic shifts in eccentricity or polar angle. The same applies to the effects of temporal filtering: can the authors exclude systematic shifts of pRF positions? 
2. I think that the figures could be more incisive if they were more compact. While I understand the importance of reporting single-participant’s data, I feel the need for gathering the data in fewer more illustrative plots. One possibility would be to use the format of scatter-plots comparing pRF parameters at 1.5 vs. 3T (so that the three subjects could be represented as different symbols within a single plot) - perhaps accompanied by a grand-average showing the expected relationship between pRF parameters and visual areas or eccentricity.
3. It might be worth pointing out that the bias introduced by temporal filtering is linked to the regular sequence of visual presentations. Presumably, temporal filtering would have had no effect on pRF size if the visual stimulus used for retinotopic mapping varied its position randomly - instead of sweeping across the visual field.
4. Sharing the raw data is sometimes very difficult, but clearly this is encouraged by many in the field. I wonder whether the authors could provide a justification for not providing .nii files (rather than the analyzed datasets, which are certainly sufficient for reproducing the figures but do not allow for reusing the dataset for novel investigations).

I confirm that I have read this submission and believe that I have an appropriate level of expertise to confirm that it is of an acceptable scientific standard.

# Response

**Published:** 2020-02-15

**Authors:** Catherine Morgan

**Affiliations:** University of Auckland, New Zealand

We’d like to thank the reviewer for their considered questions and useful suggestions on this manuscript. In our response we have reproduced the report, and commented immediately after each point.

“pRF mapping provides a quantitative description of cortical representations, including the spatial representation in visual cortical areas. While pRF parameters are extensively used to quantitatively compare across subjects and conditions, we are well aware that such parameters depend on both neural factors and on nuisance variables that are related to the dynamics of BOLD. However, we lack a precise understanding of how such nuisance variables might affect each of the pRF parameters. Morgan & Schwarzkopf make an important effort in this direction and test the impact of static SNR (affected by MR field strength) and temporal SNR (affected by temporal filtering) on spatial pRF parameters in visual cortical areas.

I have no major concerns with the study and only offer few suggestions that hopefully will increase the usability of the reported results:

    It would be useful to explicitly describe one (or more) models that predict changes of pRF parameters with SNR.

    I feel one intuitive (and probably wrong) model invokes a non-linear thresholding effect. The logic is, approximately, as follows: when BOLD responses are stronger, each voxel responds to a larger range of stimulus position and the pRF size is consequently increased. Is this one of the hypotheses that the study intends to test (and falsify)?

    This particular model would predict that beta values (indexing BOLD response amplitude) and pRF size estimates correlate positively. Since both indices were collected here, could the authors measure this (lack of) correlation?”

>>Thank you for this important question. A stronger response amplitude could potentially appear as a larger pRF because visual field locations farther from the pRF centre respond to the stimulus. However, this is unlikely to manifest as a positive correlation with the response amplitude (beta) parameter because the pRF model is scale-invariant, that is, the pRF parameters are estimated by correlation between predicted and observed time series and thus theoretically only the *pRF shape *matters to estimation.

In fact, the opposite relationship, that pRF size estimates are larger with low beta parameters, is actually more realistic: response amplitudes are linked to signal-to-noise and thus goodness-of-fit. Under low noise conditions, a small pRF would show a clean response time series with very clearly defined peaks. In a noisier pRF, this time series would however be contaminated and so the peaks of the response would be less clearly defined. In order to fit a pRF model, the algorithm will therefore produce an artifactual bias on pRF size estimates.

We have already seen this relationship in previous work. However, for the sake of completeness we now conducted the suggested correlation analysis between beta parameters and pRF size at the single-vertex level. This confirms that the second model is indeed correct: across participants and visual regions of interest there is a negative correlation between pRF size estimates and beta parameters (Spearman’s rho ranging between -0.17 and -0.5 for most cases, with the exception of V3A where the small amount of data may skew the results).

In other words, when response amplitude is low, pRFs tend to be estimated as *larger*. Unfortunately, this analysis is complicated by several factors: the data are highly heteroscedastic (there is far more variability in response amplitude for small pRFs than large ones), a small but considerable proportion of beta parameters is negative (we believe these are related to artifactual pRF estimates with negative BOLD responses due to large blood vessels or edge-of-stimulus artifacts), and there is also a strong but incomplete ceiling artifact as many pRF sizes cluster at a small size. Nevertheless, we believe due to its consistency and robustness to thresholding this relationship probably correct.

We now briefly discuss this issue in the introduction of the revised manuscript. Please note that because we find no consistent difference in pRF size between scanner sites, there is no evidence that this link has any bearing on the conclusions of our present study. Therefore, and because of their complexity, we chose not to present and discuss the results of this control analysis in the revised manuscript. However, the analysis script for this is now included in our online repository.

    “In addition, this model would incorrectly predict larger pRF size estimates at higher MR fields, under the assumption that response amplitude is positively related to SNR. Could the authors report whether this assumption (larger beta-values in the higher SNR, i.e. 3T, dataset) is correct for their specific dataset?”

>> As the reviewer correctly points out, in MRI, the signal-to-noise ratio (SNR) is directly proportional to main magnetic field strength (and voxel volume) (Edelstein, Glover, Hardy, & Redington, 1986). Therefore power to detect BOLD activation theoretically increases with static magnetic field (or larger voxel sizes), or where this is not possible/desirable, longer scan times. However, noise in fMRI data has multiple contributions (physiological, thermal and system related) and therefore the relationship between the temporal signal to noise ratio (TSNR) in a fMRI time course has a non-linear relationship with static SNR. This has been demonstrated theoretically and empirically (Murphy, Bodurka, & Bandettini, 2007) showing that any gains in static SNR, from e.g. increased field strength, will not translate to proportional gains in TSNR due to a limit where physiological noise dominates.

Since response amplitude (beta) is related to goodness-of-fit, we assume that betas are indeed greater at 3T. We now conducted the suggested analysis and this confirms that prediction: betas are larger at 3T at least in the early visual areas showing a similar pattern as goodness-of-fit. We added this figure to what is now Supplementary Figure S1.

But importantly, as we explained above, larger betas do not result in larger pRF size estimates but rather the opposite. Despite this, we found no consistent differences in pRF size estimates between the two scanner sites.

    “Also, I wonder whether one could also expect variations of the other pRF parameter (pRF position), e.g. in the form of an attraction towards the fovea / screen borders / horizontal and vertical meridians. The text reports relatively high correlations between pRF center parameters at 1.5 and 3T, but this does not exclude systematic shifts in eccentricity or polar angle. The same applies to the effects of temporal filtering: can the authors exclude systematic shifts of pRF positions? “

We would not predict any systematic relationship between pRF position and magnetic field strength but this is an interesting suggestion. There does not appear to be any strong evidence of such a systematic bias in the maps at least (please refer to the example maps we included – an interested reader may be able to reproduce them using our shared analysis scripts). The biggest differences in eccentricity maps between the two sites appear to be additional voxels within the foveal region in the 3T data, rather than an obvious shift in the map. We however understand that this visual inspection of the maps is only a very coarse analysis.

A quantitative analysis would be to plot visual field positions directly against one another. For reasons that are outside the scope of this discussion, it is imperative that this comparison must be done using an independent criterion for determining which data points we compare between the two sites. We therefore determined the cortical distance of each vertex in the spherical surface model from a point chosen near the occipital pole as a proxy of ground truth eccentricity. We then quantified the mean eccentricity within 5 mm cortical distance bins for the two sites. This revealed no obvious systematic differences in eccentricity between the two sites. We believe this analysis is too complex to be included in the main publication but we included the code for it in our public repository and made this figure publicly available: https://osf.io/k4dcr/

    “I think that the figures could be more incisive if they were more compact. While I understand the importance of reporting single-participant’s data, I feel the need for gathering the data in fewer more illustrative plots. One possibility would be to use the format of scatter-plots comparing pRF parameters at 1.5 vs. 3T (so that the three subjects could be represented as different symbols within a single plot) - perhaps accompanied by a grand-average showing the expected relationship between pRF parameters and visual areas or eccentricity.”

>> Thank you for this observation. We agree both you and the other reviewer make a good point and we have now consolidated the individual plots into group average plots, and moved the individual figures into the supplementary material.

We also like your idea of scatter plots but we believe they would also be somewhat complex as we would ideally also need to denote the different eccentricities (perhaps with a colour code). Especially for measures that do not change linearly with eccentricity (like beta or goodness-of-fit) this would make for very dense plots.

    

    It might be worth pointing out that the bias introduced by temporal filtering is linked to the regular sequence of visual presentations. Presumably, temporal filtering would have had no effect on pRF size if the visual stimulus used for retinotopic mapping varied its position randomly - instead of sweeping across the visual field.

>> This is a very good suggestion and we now discuss this in the revised manuscript. However, please also keep in mind that other work by ourselves ( ^1^, currently under peer review, but a preprint is available here: https://doi.org/10.1101/821918) and of course also your own work ^2^has shown that pRF size estimates differ between random and ordered designs. This issue was also discussed in a study on tonotopic pRF mapping ^3^. It is possible that temporal filtering would not show the effect we report here for a random design but there are probably additional factors linking pRF size with randomisation. For instance, we showed in Infanti & Schwarzkopf that whether or not pRF size is over- or underestimated in ordered relative to random designs also depends on the stimulus parameters.

    Sharing the raw data is sometimes very difficult, but clearly this is encouraged by many in the field. I wonder whether the authors could provide a justification for not providing .nii files (rather than the analyzed datasets, which are certainly sufficient for reproducing the figures but do not allow for reusing the dataset for novel investigations).

>> please refer to our response to reviewer 1 to answer this point. In short, there are both legal and ethical barriers to our sharing of raw data files. However, we want to stress that all of the time series information necessary for reproducing the pRF analysis, reanalysing it with a different software, or conducting additional novel analyses is available in our public repository. For anyone seeking to conduct analysis that are not possible with the shared data we are willing to make these data available upon request provided the participants consent to this use.

Response References

1.         Infanti, E. & Schwarzkopf, D. S. Mapping sequences can bias population receptive field estimates. *bioRxiv* 821918 (2019) doi:10.1101/821918.

2.         Binda, P., Thomas, J. M., Boynton, G. M. & Fine, I. Minimizing biases in estimating the reorganization of human visual areas with BOLD retinotopic mapping. *J. Vis.* **13**, 13–13 (2013).

3.         Thomas, J. M. *et al.* Population receptive field estimates of human auditory cortex. *NeuroImage* **105**, 428–439 (2015).

# Peer review: Reviewer response for version 1

**DOI:** 10.5256/f1000research.22528.r54262

**Published:** 2019-10-25

**Authors:** Noah Benson

**Affiliations:** 1 Department of Psychology, Center for Neural Science, New York University, New York, NY, USA

Population receptive field (pRF) modeling is an incredibly important and common technique in visual and perceptual neuroscience. PRF mapping is also complex, and, despite its popularity, few examinations of how the many incidental complexities of collecting pRF maps affect the maps themselves have been reported. This lack makes comparison and interpretation of pRF results across sites and studies difficult. In this article, Morgan and Schwarzkopf provide a straightforward examination of two of these incidental complexities of retinotopic pRF mapping: the field-strength of the scanner and the choice to low-pass filter the BOLD time-series. Encouragingly, they find that the effect of the field-strength is minimal; however, the effects of the filtering are non-trivial.

 I have no major concerns regarding the methods of this paper, but I do have a few suggestions. First, it is not clear if the authors used the same hemodynamic response function (HRF) for all subjects or if a single canonical function was fit to empirical data from each subject individually, nor do the authors explain which canonical HRF was used; I would like to see the HRF form(s) reported explicitly. Secondly, the use of linearly-spaced eccentricity bands for the various further analyses is a somewhat questionable choice given that the number of vertices between 8.5° and 9.5° will be substantially smaller than the number of vertices between 0.5° and 1.5°. This effect may partly explain why the errors in some figures (e.g. Figure 6) are quite large at higher eccentricities. A principled way to obtain similarly-sized eccentricity bins might be to use Horton and Hoyt's 1991 equation ^1^ for cortical magnification [CMF( *e*) = (17.3 / (0.75 + *e*)) ^2^ (mm/deg) ^2^ for eccentricity *e*], integrate it over the visual field to obtain an estimate of cortical surface area in terms of eccentricity [SAF( *e*) (mm ^2^)], then to invert this [SAF ^-1^( *a*) (deg) for cortical surface area *a*] and choose eccentricity values that correspond to values of SAF ^-1^( *a*) for linearly-spaced surface-area values of *a*.

 Despite the above suggestions, the results of the paper are quite clear: (1) pRF models are robust to changes in the field strength of the scanner, and (2) temporal filtering inflates the *R* ^2^ and size parameter of the pRF fits without substantially changing the topographic structure of the retinotopic maps. Both of these insights are useful additions to the field, especially given the rapid rate of change in MRI technology and the frequency with which temporal smoothing is applied to pRF mapping data. More work is needed throughout the field to quantify these kinds of effects, and this paper is a useful contribution to that effort. One minor suggestion is that the authors might explicitly state in the discussion that the two scanners share a manufacturer; this is both a limitation of the comparison, in that the results may not generalize across manufacturers, and one of its strengths, in that one can be more confident of the findings with respect to field-strength because the manufacturer was not an additional variable across sites.

 My biggest concerns about the paper center on the supplemental data. Although the authors have provided enough data to reproduce the figures and aspects of the study, the data provided are incomplete and fail to conform to contemporary standards of reusability and interoperability. For one, all of the supplemental data files could have been provided in well established community-vetted formats (such as NIFTI files), yet *none* of the data are provided in such formats (in fact many of the files cannot be easily read without an expensive MATLAB license). Additionally, only some of the data are provided: FreeSurfer reconstructions, anonymized anatomical images, and various raw data are not provided. These data really ought to be part of the dataset, and ideally should be provided in accordance with the BIDS spec ^2^. A few examples of things that cannot be examined with the current dataset: concerns about field distortions, questions about partial voluming artifacts, concerns about signal attenuation near vessels, questions about how the results relate to the surface area of the visual areas, and questions about how the results generalize to frontal cortex. To be clear, none of these examples are likely to impact the conclusions of the study, but I point them out as examples of how the current dataset fails to meet the evolving standards of data-availability in the field.

 Beyond these comments, I have only minor concerns that are largely aesthetic in nature:

 The use of the prime symbol (′) and the single quote (') is inconsistent and, due to the occasional adjacency of variable symbols with subscripts next to commas, can be a bit confusing.

 Figures 3, 4, and 6 contain a lot of information, and I feel that a panel that summarizes or combines the data across subjects in some way would be useful and easier to interpret.

 The eccentricity colormap used in Figure 2 is circular, which causes some ambiguity between very different values.

 Figure 1 seems to have a larger range (along the y axis) for the filtered data than for the unfiltered data. It is not clear to me how the filtering described would produce a signal with nearly twice the range of values as the original signal. This might be a plotting error.

I confirm that I have read this submission and believe that I have an appropriate level of expertise to confirm that it is of an acceptable scientific standard.

## References

[1] : The representation of the visual field in human striate cortex. A revision of the classic Holmes map. *Arch Ophthalmol*.1991;109(6) : 10.1001/archopht.1991.01080060080030 816-24 10.1001/archopht.1991.01080060080030 2043069

[2] : The brain imaging data structure, a format for organizing and describing outputs of neuroimaging experiments. *Sci Data*.2016;3: 10.1038/sdata.2016.44 160044 10.1038/sdata.2016.44 27326542PMC4978148

# Response

**Published:** 2020-02-15

**Authors:** Catherine Morgan

**Affiliations:** University of Auckland, New Zealand

We’d like to thank the reviewer for their considered questions and useful suggestions on this manuscript. In our response we have reproduced the report, and commented immediately after each point.

 “Population receptive field (pRF) modeling is an incredibly important and common technique in visual and perceptual neuroscience. PRF mapping is also complex, and, despite its popularity, few examinations of how the many incidental complexities of collecting pRF maps affect the maps themselves have been reported. This lack makes comparison and interpretation of pRF results across sites and studies difficult. In this article, Morgan and Schwarzkopf provide a straightforward examination of two of these incidental complexities of retinotopic pRF mapping: the field-strength of the scanner and the choice to low-pass filter the BOLD time-series. Encouragingly, they find that the effect of the field-strength is minimal; however, the effects of the filtering are non-trivial.

 I have no major concerns regarding the methods of this paper, but I do have a few suggestions. First, it is not clear if the authors used the same hemodynamic response function (HRF) for all subjects or if a single canonical function was fit to empirical data from each subject individually, nor do the authors explain which canonical HRF was used; I would like to see the HRF form(s) reported explicitly.”

 >> For all subjects we used the same canonical HRF based on previous data (de Haas et al. 2014). The reviewer is right that the HRF used can have an effect on the obtained pRF parameters but this effect is typically very small (Dumoulin & Wandell, 2008). In our previous study quantifying the test-retest reliability of pRF parameters on the same scanner we found only minimal effects of obtaining subject-specific HRF fits. While it is indeed possible that the HRF differs between our scanners in the present study, this effect is also unlikely to alter our conclusions considering the already very similar pRF parameters we observed. We thank you for pointing this ambiguity out and will edit the manuscript accordingly.

 “Secondly, the use of linearly-spaced eccentricity bands for the various further analyses is a somewhat questionable choice given that the number of vertices between 8.5° and 9.5° will be substantially smaller than the number of vertices between 0.5° and 1.5°. This effect may partly explain why the errors in some figures (e.g. Figure 6) are quite large at higher eccentricities. A principled way to obtain similarly-sized eccentricity bins might be to use Horton and Hoyt's 1991 equation 1 for cortical magnification [CMF( *e*) = (17.3 / (0.75 + *e*)) ^2^ (mm/deg) ^2^ for eccentricity *e*], integrate it over the visual field to obtain an estimate of cortical surface area in terms of eccentricity [SAF( *e*) (mm ^2^)], then to invert this [SAF ^-1^( *a*) (deg) for cortical surface area *a*] and choose eccentricity values that correspond to values of SAF ^-1^( *a*) for linearly-spaced surface-area values of *a*.”

 >> The reviewer raises a very important point and we have previously debated this in the lab and also with other researchers. Linearly scaled eccentricity bands is the standard procedure used by most pRF studies . It is true that a logarithmic scaling of eccentricity bands would ensure approximately equal amounts of data per eccentricity band, and thus ensure relatively even error margins. It however seems to us unlikely that this will alter our conclusions in any way or that this will reveal additional information. Practically, it would reduce the number of bins which could also be suboptimal for comparing the findings by visual inspection. As an example, we included the equivalent to the figure for the pRF size from the two scanning sites but using logarithmic bands online: https://osf.io/nyj4z/

 To our eyes this does not alter what we can interpret from this plot. Note also that the most peripheral band still contains less data because edge artifacts are not always consistent and thus we cannot ensure equal surface area for this band. We therefore decided to go with tradition and stick with linear bins.

 “Despite the above suggestions, the results of the paper are quite clear: (1) pRF models are robust to changes in the field strength of the scanner, and (2) temporal filtering inflates the *R* ^2^ and size parameter of the pRF fits without substantially changing the topographic structure of the retinotopic maps. Both of these insights are useful additions to the field, especially given the rapid rate of change in MRI technology and the frequency with which temporal smoothing is applied to pRF mapping data. More work is needed throughout the field to quantify these kinds of effects, and this paper is a useful contribution to that effort. One minor suggestion is that the authors might explicitly state in the discussion that the two scanners share a manufacturer; this is both a limitation of the comparison, in that the results may not generalize across manufacturers, and one of its strengths, in that one can be more confident of the findings with respect to field-strength because the manufacturer was not an additional variable across sites.”

 >> We’d like to thank the reviewer for highlighting this very important point and will add it to the discussion.

 “My biggest concerns about the paper center on the supplemental data. Although the authors have provided enough data to reproduce the figures and aspects of the study, the data provided are incomplete and fail to conform to contemporary standards of reusability and interoperability. For one, all of the supplemental data files could have been provided in well established community-vetted formats (such as NIFTI files), yet *none* of the data are provided in such formats (in fact many of the files cannot be easily read without an expensive MATLAB license). Additionally, only some of the data are provided: FreeSurfer reconstructions, anonymized anatomical images, and various raw data are not provided. These data really ought to be part of the dataset, and ideally should be provided in accordance with the BIDS spec 2. A few examples of things that cannot be examined with the current dataset: concerns about field distortions, questions about partial voluming artifacts, concerns about signal attenuation near vessels, questions about how the results relate to the surface area of the visual areas, and questions about how the results generalize to frontal cortex. To be clear, none of these examples are likely to impact the conclusions of the study, but I point them out as examples of how the current dataset fails to meet the evolving standards of data-availability in the field.”

 >> We are very supportive of the idea of open science in general, for data integrity and reproducibility. However, data transparency must be traded off against ethical and data protection concerns. Any brain imaging data is by nature not fully de-identifiable, especially if it contains anatomical/morphological information. This is a particular issue with a small sample size like this (which is not uncommon for retinotopic mapping studies). Moreover

 we also have reason to believe that under New Zealand laws (specifically the *Privacy Act (1994)* and the Health Information Privacy Code (HIPC); a set of legally-binding rules for applying this act) we cannot share our full imaging data (including volumetric EPI data and FreeSurfer reconstructions) publicly without any custodianship as it can only be shared for research and for the same purpose as our original ethical approval. We are currently in the process of seeking legal guidance on this point and determine a consistent policy on data sharing within our institution.

 However, even in the absence of all the raw data, the data we do share are fully transparent and theoretically permit anyone to reproduce our findings. As opposed to the reviewer’s concern, our data do not require a MATLAB license but can be read using the free software Octave and FreeSurfer. Octave misses some of the functionality of MATLAB and we are currently working on opening this up but critically the data are fully accessible. All the pre-processed time series for individual voxels/vertices are also included which would allow an interested research to carry out their own pRF analysis on these data.

 Regarding the format, at present we always share our data in the MATLAB format because that is what our SamSrf toolbox uses. It is convenient because it allows us to store all the functional data per hemisphere in one file, in a relatively straightforward data structure, with maps and time series information of all vertices of the cortical reconstruction in a single 2D matrix. We however see the merit in a ready exchange of data between different platforms and are currently considering and debating adding support for standard formats that allow this, but in our view this is outside the scope of this revision..

 “Beyond these comments, I have only minor concerns that are largely aesthetic in nature:

 The use of the prime symbol (′) and the single quote (') is inconsistent and, due to the occasional adjacency of variable symbols with subscripts next to commas, can be a bit confusing.”

 >> We thank the reviewer for pointing this out and will change this in our revision.

 “Figures 3, 4, and 6 contain a lot of information, and I feel that a panel that summarizes or combines the data across subjects in some way would be useful and easier to interpret.”

 >> This is an interesting point. Considering our sample size is 3 we believe presenting individual data is important. However, the group results are also very clear and we therefore decided to present only group averages in the main manuscript now, thank you for the suggestion, and instead relegate the individual plots to the Supplementary Information.

 “The eccentricity colormap used in Figure 2 is circular, which causes some ambiguity between very different values.”

 >> The choice of the same colour scheme for polar angle and eccentricity is in part a leftover from old days in which eccentricity maps were based on phase-encoded analysis. We agree that this could be somewhat confusing, although our rendering ensures that this is not the case. The fovea is represented by red whereas the most peripheral eccentricities are shown in purple. The colour scheme *does not cycle back*. Instead, all eccentricities above the outer edge of the stimulus (9.5 degrees) are set to purple.

 “Figure 1 seems to have a larger range (along the y axis) for the filtered data than for the unfiltered data. It is not clear to me how the filtering described would produce a signal with nearly twice the range of values as the original signal. This might be a plotting error.”

 >> We thank the reviewer for pointing this out. This is an artifact in this illustration and the Y-axis should really be in arbitrary units. We will change this in our revision.

 >> Response References

 de Haas B, Schwarzkopf DS, Anderson EJ, et al.: Perceptual load affects spatial tuning of neuronal populations in human early visual cortex. Curr Biol. 2014; 24(2): R66–67.              

 Dumoulin SO, Wandell BA: Population receptive  eld estimates in human visual cortex. NeuroImage. 2008; 39(2): 647–660.
